# Characterisation of community acquired non-typhoidal Salmonella from bacteraemia and diarrhoeal infections in children admitted to hospital in Nairobi, Kenya

**DOI:** 10.1186/1471-2180-6-101

**Published:** 2006-12-15

**Authors:** Samuel Kariuki, Gunturu Revathi, Nyambura Kariuki, John Kiiru, Joyce Mwituria, Charles A Hart

**Affiliations:** 1 Centre for Microbiology Research, Kenya Medical Research Institute, Nairobi, Kenya; 2 Department of Medical Microbiology, Kenyatta National Hospital, Nairobi, Kenya; 3 Department of Paediatrics and Child Health, University of Nairobi, Kenya; 4 Department of Medical Microbiology and Genito-Urinary Medicine, University of Liverpool, Liverpool, UK

## Abstract

**Background:**

In sub-Saharan Africa community-acquired non-typhoidal Salmonella (NTS) is a major cause of high morbidity and death among children under 5 years of age especially from resource poor settings. The emergence of multidrug resistance is a major challenge in treatment of life threatening invasive NTS infections in these settings.

**Results:**

Overall 170 (51.2%) of children presented with bacteraemia alone, 28 (8.4%) with gastroenteritis and bacteraemia and 134 (40.4%) with gastroenteritis alone. NTS serotypes obtained from all the cases included *S*. Typhimurium (196; 59%), *S*. Enteritidis (94; 28.3%) and other serotypes in smaller numbers (42; 12.7%); distribution of these serotypes among cases with bacteremia or gastroenteritis was not significantly different. A significantly higher proportion of younger children (< 3 years of age) and those from the slums presented with invasive NTS compared to older children and those from upper socio-economic groups (p < 0.001). One hundred and forty-seven (44.3%) NTS were resistant to 3 or more antibiotics, and out of these 59% were resistant to ampicillin, chloramphenicol and tetracycline. There was no significant difference in antibiotic resistance between the two serotypes, *S*. Typhimurium and *S*. Enteritidis. Ceftriaxone and ciprofloxacin were the only antibiotics tested to which all the NTS were fully susceptible. Using Pulsed Field Gel Electrophoresis (PFGE) there were 3 main patterns of *S*. Typhimurium and 2 main patterns of *S*. Enteritidis among cases of bacteraemia and gastroenteritis.

**Conclusion:**

Serotype distribution, antibiotic susceptibility and PFGE patterns of NTS causing bacteraemia and gastroenteritis did not differ significantly. The high prevalence of NTS strains resistant to most of the commonly used antimicrobials is of major public health concern.

## Background

In many sub-Saharan African countries community-acquired bacteraemia is a major cause of high morbidity and death among children especially from resource poor settings. Non-typhoidal salmonellae (NTS) account for a steadily increasing proportion of these infections and represent from 20–50% of cases [1-3]. In Kenya invasive NTS infections in children under 5 years of age are an important cause of morbidity and high mortality; they are ranked second only to pneumococcal pneumonia in importance as the leading bacterial cause of child mortality [4,5]. In contrast in industrialized countries the most common manifestation of NTS infection is gastroenteritis, which is usually a self-limiting and benign disease, invasion beyond the gastrointestinal tract occurs in only approximately 5% of patients with salmonellosis, even among children [6,7]. In this small proportion of children from industrialized countries with NTS bacteraemia, the infection usually occurs in previously healthy young children, it appears in the context of gastroenteritis, and has a favourable outcome [8,9]. However, lack of adequate diagnostic capabilities in poor resource settings that is prevalent in most public health facilities in Africa may hinder prompt diagnosis of infection and may often lead to under-diagnosis, delay appropriate treatment and often result in poor prognostic outcomes compared to similar cases in industrialized countries.

Previous studies in Kenya reported on characteristics of bacteremic cases of NTS in children [4,5,10], but no study has compared presentations of NTS infections in gastroenteritis and bacteraemia, and their clinical and bacteriological characteristics. We report on a study that prospectively investigated children admitted to hospital with NTS bacteraemia and gastroenteritis in an urban setting, the antimicrobial susceptibility patterns and the genotypic characteristics of NTS strains causing these infections.

## Results

### Demographics

A total of 332 children aged between 4 weeks and 84 months (median age 24 months) reporting to hospital with either NTS gastroenteritis or bacteraemia were recruited into the study. There were more girls than boys (184 vs 148), but the difference was not significant (p > 0.05). The majority of cases (72.2%) were within the age group less than 3 years compared to the age group above 3 years (p < 0.001) and with a peak in cases occurring within the age group 1–3 years. More than a third (128; 38.6%) of the children with NTS isolations came from three main slum areas of the city with a catchment of approximately 100,000 children under 3 years of age. Another 123 (37%) of the children came from the middle income class, while another 24.4% of the children came from upper class population. Two thirds of the patients from the upper class group were seen at the private hospital, while 105 (82%) children from the slum areas and 76 (61.8%) children from middle income class were seen at the public hospital.

A total of 91% of all parents/caregivers interviewed said their homes had access to treated tap water either within their household (75%) or from a community watering point (25%), but there were occasional shortages which forced them to store water in tanks at home for days or weeks. Only 3–5% of those interviewed particularly from the periurban areas around the city used boreholes, wells or rivers near their homes to obtain water for domestic use.

**Figure 1:**
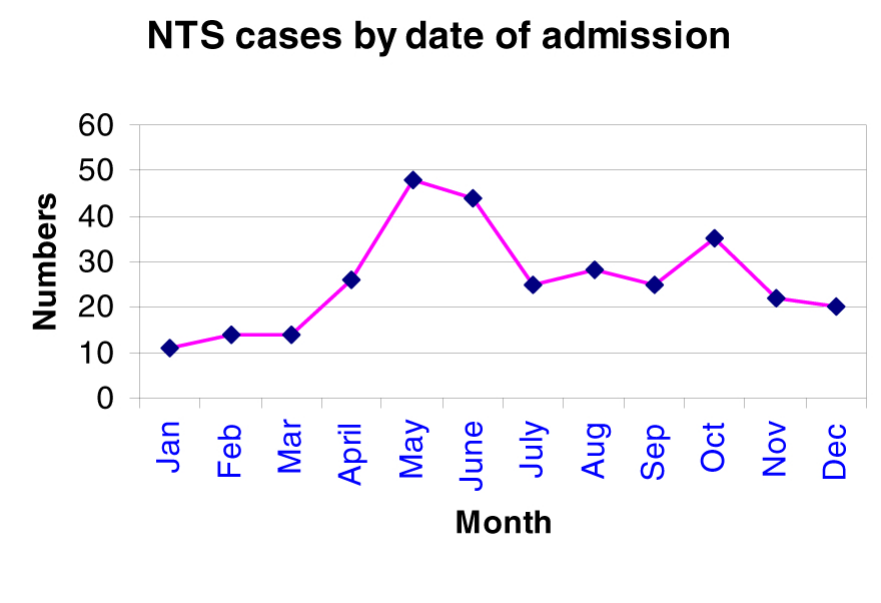
Number of NTS isolates from children by month. A higher number of isolations occurred during the rainy season in May and June compared to the rest of the year (p < 0.001).

A monthly average of 46 admissions was recorded for all NTS infections during the rainy months of May and June, which was significantly higher than an average of 22 monthly admissions recorded for the rest of the year when it was generally dry or with lighter rains (< 10 mm) (p < 0.001) (Figure 1). There was no significant difference in the NTS serotype distribution between isolates from diarrhoeal or from the bacteremic cases during the two main rainy seasons of the year.

### Clinical presentation of NTS bacteraemia and gastroenteritis

Overall 170 (51.2%) of children presented with bacteraemia alone, 28 (8.4%) with gastroenteritis and bacteraemia and 134 (40.4%) with gastroenteritis alone (Table 1). For both bacteremic and diarrhoeal NTS infections, most of the cases occurred within the age group 1–3 years (p < 0.001). As would be expected a significantly higher proportion of children with NTS bacteraemia presented with fever compared to those that had gastroenteritis only [117/198 (61.8%) versus 37/134 (27.6%), p < 0.001]. In contrast, a higher proportion of children with gastroenteritis reported with symptoms of diarrhoea and vomiting [89 (66.4%) and 66 (49.3%) respectively] compared to children who had bacteraemia [95 (48%) and 46 (23.2%) respectively] and the difference was statistically significant (p < 0.001). NTS serotypes obtained from all the cases included *S*. Typhimurium (196; 59%), *S*. Enteritidis (94; 28.3%) and other serotypes consisting of *S*. Haifa, *S*. Braenderup, *S*. Choleraesuis, *S*. Dublin, *S*. StPaul and *S*. Indiana in smaller numbers (42; 12.7%). For all the age groups, the isolation rate for *S*. Typhimurium was nearly double or more that of *S*. Enteritidis – the 2 most common serotypes in bacteraemia and gastroenteritis. For serotype *S*. Typhimurium there was a higher proportion of isolates from cases with gastroenteritis only (92; 68.7%) compared to those who presented with NTS bacteraemia alone (93; 52.5%) (p = 0.04).

**Figure 2:**
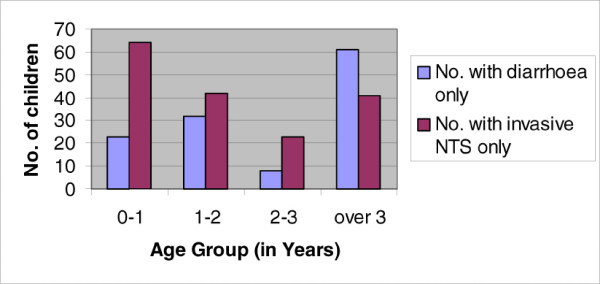
Clinical presentation of NTS infection according to age group. More children under the age of 3 years tended to get invasive disease compared to older children (over 3 years) who had a higher proportion with gastroenteritis.

A significantly larger proportion of invasive NTS were obtained from children under 3 years of age compared to older children (p < 0.001) (Figure 2). In addition, a significantly higher proportion of invasive NTS were obtained from children from the slum areas compared to children from middle income population and the upper socieconomic class (p < 0.001). In contrast, a higher proportion of non-invasive NTS were obtained from children from the upper socieconomic class compared to children from the slum areas or the middle income group (p < 0.001) (Table 2). The length of hospitalisation for children with NTS bacteraemia was 1–60 days and 1–20 days for children admitted with gastroenteritis only. The median period of hospitalisation for NTS bacteremic cases was significantly longer than for NTS gastroenteritis alone (9 days versus 2 days) (p = 0.004). Overall, only 26 (7.8%) of patients admitted had a diagnosis of malaria confirmed using a malaria slide test, and more than half of these were children with NTS bacteraemia. Other key concomitant infections in paediatric patients admitted with NTS infection were rotavirus diarrhoea (40; 12%) (36/40 of the cases occurred in patients with NTS in stool) and *Entamoeba histolytica *infection (15; 4.5%). However, there was no significant association between NTS presentation and presence of concomitant infections.

### Antibiotic susceptibility of NTS from bacteremic and diarrhoeal cases

NTS from both blood and stool showed a high level of resistance to commonly used antibiotics including ampicillin, cefuroxime and cotrimoxazole (Table 3). For both bacteremic and diarrhoeal cases, NTS from the age group <3 years showed the highest level of resistance to all the commonly used antibiotics (p = 0.001). A total of 147 NTS were multidrug resistant (resistant to 3 or more antibiotics), and out of these 59% were resistant to ampicillin, chloramphenicol and tetracycline. For MDR NTS the highest level of resistance to commonly used antibiotics including ampicillin, chloramphenicol and tetracycline was for infections within the age group less than 3 years compared to older children (p < 0.001). However for gentamicin, tetracycline and nalidixic acid, NTS from bacteremic cases showed significantly higher resistance levels than NTS from diarrhoeal cases (p = 0.001). Co-amoxiclav was the only antibiotic to which NTS from diarrhoeal cases showed significantly higher resistance to than NTS from bacteremic cases (p = 0.0012). There was no significant difference in susceptibility to antibiotics between the two main serotypes causing illness in the children. No NTS isolates were observed to produce extended spectrum beta-lactamases. Ceftriaxone and ciprofloxacin were the only two antibiotics to which all the NTS were fully susceptible.

### Antibiotic treatment for NTS and clinical outcomes

Treatment data were available for only 241 (72.5%) of the patients (Table 4). Majority of cases of NTS infections in both hospitals were treated using cefuroxime (32%) for both gastroenteritis and bacteraemia and ceftriaxone if the case was life threatening bacteremic infection. In addition, gentamicin and co-amoxiclav were also popular with paediatricians in the referral hospital due to their lower costs. As can be seen in Tables 3 and 4 resistance data indicate that 30% of patients treated with cefuroxime were likely to fail in their treatment. The level of resistance was much higher for ampicillin and cotrimoxazole, but fortunately fewer paediatricians used these antibiotics for treatment of NTS infections. Ceftriaxone was the only antibiotic used to which NTS were fully susceptible. However, this antibiotic is too expensive particularly for patients in public hospitals in rural areas of the country. At the National Referral Hospital available clinical data showed that 35% of all NTS bacteremic cases in newborns and those below 4 weeks of age and 12% of those between 1 and 3 years of age died despite being given optimal care. For children above 5 years of age mortality was < 3%. The mortality rate was 5% and < 1% for age groups below 4 weeks and 1–3 years, respectively at the private hospital.

### Genotypic relationship of NTS strains by PFGE

**Figure 3:**
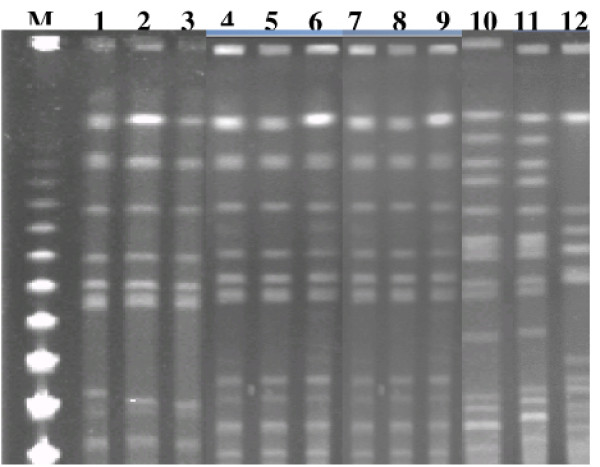
Representative PFGE patterns of *Xba*I-digested *S*. Typhimurium from cases from children with bacteraemia. Lane M, 50 kb molecular size standard; Lanes 1 to 9, *S*. Typhimurium from PFGE pattern 1; Lanes 10 and 11, *S*. Typhimurium from PFGE pattern 2; Lane 12 *S*. Typhimurium from PFGE pattern 3. Using either of the two enzymes, *Xba*I or *Spe*I, PFGE patterns of the isolates were within 1–3 band differences.

**Figure 4:**
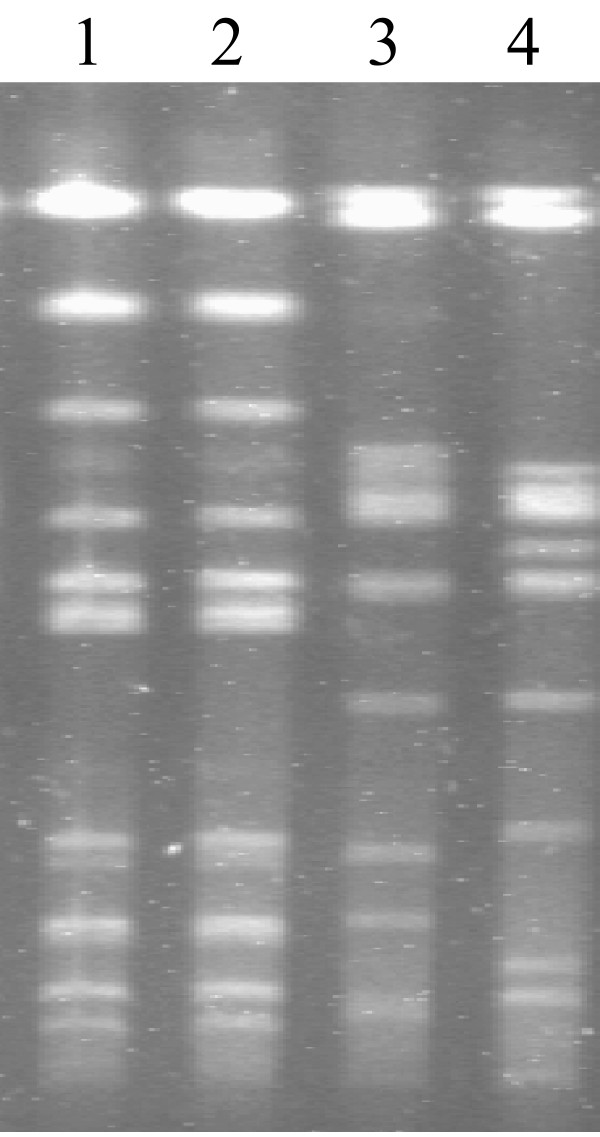
Representative PFGE patterns of *Xba*I-digested *S*. Enteritidis from cases from children with bacteraemia. Lane M, 50 kb molecular size standard. Lanes 1 and 2, *S*. Enteritidis from PFGE pattern 1; Lanes 3 and 4, *S*. Enteritidis from PFGE pattern 2.

PFGE electrophoresis of *Xba*I and *Spe*I was performed for the two most common NTS serotypes; *S*. Typhimurium and *S*. Enteritidis. There were 3 main genotypes of *S*. Typhimurium and 2 main genotypes of *S*. Enteritidis. There was no significant difference in the distribution of NTS genotypes among cases of bacteraemia or gastroenteritis. In addition there was no significant association between resistance profiles and PFGE patterns observed (Figure 3 and Figure 4).

## Discussion

Several studies in tropical Africa investigating invasive diseases in children have shown that NTS are important causes of life threatening infection particularly in infants and young children below the age of 5 years. The most common manifestation of invasive NTS is bacteraemia, followed by meningitis, osteomyelitis, endocarditis and arthritis [3,5,11]. In contrast, studies from most parts of the developed world found that most NTS infections in children presented as gastroenteritis and the reported incidence of bacteraemia among children with gastroenteritis caused by NTS varies between 3.3% and 41% [12-15], although majority of studies reported incidences at no more than 5%. In our study 59.6% of NTS cases in paediatric admissions were invasive compared to 39.8% NTS cases that presented with gastroenteritis only (p < 0.01). Although not statistically significant a higher proportion of children under 3 years of age (as compared with children over 3 years) presenting with NTS infection had invasive infection and this may be attributed to lower immune status in the younger children. Although our study did not have access to data on HIV and nutritional status of the children, previous studies on children with bacteraemia showed that HIV, young age and malnutrition were independently associated with bacteraemia, but there was no evidence of an interaction between the effects of HIV infection and malnutrition [5]. In addition to these factors, co-morbidity with malaria may play a significant role in prolonging hospital stay for children with blood stream infection in Kenya.

More than a third (37.8%) of the children with NTS admissions came from the three main slum areas of the city. A significantly higher proportion of this population of children reported to hospital with invasive NTS infections compared to the other two socioeconomic groups (p < 0.001). In addition for the age group 1 – 84 months, higher mortality rates (12%) were recorded for children admitted to the public hospitals (and the majority were from the slums) compared to those seen at the private hospital (< 1%) (p < 0.001). It is probable that children from slum areas, for socioeconomic reasons, are less likely to present unless they have severe (invasive) disease and this would provide a bias in interpreting our data. However, our data compares well with that of Berkley *et al*., [5] who observed that of all admissions with febrile illness in a rural District Hospital, NTS constituted 18% of detected bacteraemias and resulted in 28% mortality compared to 5.7% mortality in children who did not have bacteraemia (p < 0.001). As has been previously documented children from the slum areas tend to suffer more malnutrition and hence are at higher risk of developing invasive disease and often with poor prognosis [5].

In the current study two main *Salmonella enterica *serotypes were isolated from cases of NTS bacteraemia and gastroenteritis, *S*. Typhimurium and *S*. Enteritidis, with the former being isolated at rates 1.5–2 times that of *S*. Enteritidis. Previous studies in Kenya on NTS bacteraemia have reported similar trends in prevalence of these two main *Salmonella *serotypes (10). Recent data from Malawi also showed that NTS were the most common blood culture isolates (40%), and NTS bacteraemia was diagnosed in 299 children during a 2-year period with a case fatality rate of 24%. *S*. Typhimurium and *S*. Enteritidis accounted for 80 and 13% of cases, respectively [1]. However studies elsewhere in developed countries [16] that reviewed the clinical course of children with NTS bacteraemia compared to that of children with gastroenteritis alone did not demonstrate any difference in clinical characteristics or outcome between these two groups.

In studies in the USA comparing antibiotic resistance and effect on hospitalisation [17] it was shown that patients infected with resistant isolates were slightly more likely to be hospitalized than were patients infected with fully susceptible isolates. Although in our study children with NTS bacteraemia had longer hospital stay (median of 9 days) and tended to have more prolonged high fever compared to children who presented with gastroenteritis only (p < 0.01), there was no significant association between antibiotic resistance levels and presentation of NTS infection for commonly available antibiotics including ampicillin, cotrimoxazole and chloramphenicol. However for gentamicin, tetracycline and nalidixic acid, NTS from bacteremic cases showed significantly higher resistance levels than NTS from diarrhoeal cases (p < 0.001). Although gentamicin in combination with amoxicillin was one of the drugs used for empiric treatment of NTS and other bacterial infections in children, tetracycline and nalidixic acid were not used and resistance may have been acquired from use in the adult population. It is noteworthy that amoxicillin and gentamicin combination was in the Government-funded public hospitals mainly for economic reasons and may not be a the optimum treatment of choice for salmonellsois. In agreement with our findings, other studies done in Israel [18] and Thailand [19] have also shown that bacteremic patients had higher fever and prolonged hospital stay compared to patients with NTS gastroenteritis.

NTS from the age group <3 years showed the highest level of resistance to all the commonly used antibiotics. Although we were unable to obtain data on the use of antibiotics without prescription in the community, a general survey indicated that sale of commonly available antibiotics such as ampicillin, cotrimoxazole, chloramphenicol over the counter without prescription and mainly for use in treatment of acute respiratory infections and childhood diarrhoea was common in the urban population. The most commonly used antibiotic for treatment of NTS infections at the hospitals was cefuroxime or a combination of ampicillin and gentamicin. The high level of usage of orally administered ampicillin and cefuroxime in the clinics and health centres and their availability over-the-counter in some chemist shops in urban centres was likely to be related to high levels of resistance observed. In addition the high levels of resistance to cotrimoxazole could be attributed to the widespread use of sulphamethoxazole-pyrimethamine (SP) for malaria treatment, which has previously been associated with increased resistance to cotrimoxazole [20]. Although it is well established that antimicrobial treatment of this condition does not add value and may even prolong carriage of NTS in patient, it is a common practice in Kenya to prescribe an antibiotic in almost all cases gastroenteritis due to belief that this may prevent progression into the bloodstream, thus leading to massive antibiotic misuse. The high levels of tetracycline resistance were unusual as this antibiotic was not used in paediatrics in the country; it is plausible that resistance may be related to use in the adult population. Other studies have also demonstrated a clear relationship between antibiotic usage and the inevitable rise in resistance especially among gut-related pathogens [21,22], thus emphasizing the need for prudent use of antibiotics which in several studies has been shown to delay emergence and minimise resistance levels to antibiotics [23,24]. Previous studies in Kenya [25] showed that tetracycline was commonly used among poultry farmers and this may also contribute to the overall resistance pool in he community. However, usage of other commonly available classes of antimicrobials in livestock farming in Kenya was minimal and the corresponding resistance levels of *E. coli *isolates from animals were less than 10% for ampicillin, chloramphenicol, gentamicin, nalidixic acid and cotrimoxazole.

Using PFGE, there were three main genotypes of *S*. Typhimurium and 2 main genotypes of *S*. Enteritidis causing both invasive and non-invasive disease in children. Our study did not observe any significant difference between genotypes of NTS causing bacteraemia and those causing gastroenteritis. In addition there was no significant difference in resistance to commonly available antimicrobials among the two main serotypes of NTS. It appears that other inherent characteristics in NTS other than strain types may be responsible for invasiveness in our serotypes. However, it must be pointed out that PFGE has a limited discriminatory power in subtyping *S*. Enteritidis and this may account for the few subtypes obtained in the analysis.

A major limitation of our study was that it was hospital based and hence biased towards children with invasive disease and may therefore not present a true reflection of incidence of diarrhoeal and bacteraemic NTS in the community. In resource poor settings most children attend local clinics that may be deficient in diagnostic facilities and it is probable that there is a large proportion of undiagnosed non-invasive and less severe NTS infections. The children that end up in the referral hospital may be those that have more severe NTS infections, or delayed visiting hospital early due to inadequate healthcare nearby, thus presenting a disproportionate bias in the number of children diagnosed in the facility with invasive illness. This bias in children presenting to referral hospital with more severe illness may also account for the apparent higher invasiveness of NTS in children from Kenya and subsequent poorer treatment outcome when compared to similar age groups with NTS in industrialised countries. It is likely that we have a large burden of undetected bacteraemia with additional attributable mortality.

In addition at the community level we have no information on what is the total number of children with diarrhoea and the actual number that progressed to bacteraemia since we have not studied the community as a whole. We just picked all positive isolations in the laboratories for patients that presented to hospital. This also presents a limitation in interpretation of our data.

## Conclusion

Serotype distribution patterns, antibiotic susceptibility patterns for commonly used antibiotics and genotype patterns of NTS causing bacteraemia and gastroenteritis did not differ significantly and differences in invasiveness between NTS in Kenya and those in developed countries may lay in inherent virulence characteristics of these bacteria. It will be important to determine what genetic factors would be specific to these invasive NTS. In addition a high proportion of NTS were multiply resistant to commonly available antibiotics and this poses a major challenge in management of NTS infections particularly in poor resource settings.

## Methods

### Study population

This was a 3-year prospective observational study. Between March 2002 and May 2005, all paediatric outpatients or admissions to a tertiary referral hospital (the paediatric ward has a 350-bed capacity) and a private hospital in Nairobi (paediatric ward capacity of 40 beds), whose blood cultures and stool cultures were positive for NTS were enrolled in the study. Blood cultures were performed on all febrile patients as a rule before any antibiotic treatment commenced. In addition total blood count and malaria slide for microscopy were done. Stool cultures were performed only when there was a presenting complaint of diarrhoea and abdominal pain. However, for all cases with gastroenteritis presenting at the outpatient department stool microscopy for leucocytes and red blood cells, ova and cysts, and rotavirus and adenovirus antigen tests were performed as a routine. If a patient presented with diarrhoea accompanied by high fever or high fever with a preceding watery loose motions both blood and stool cultures were done. The following criteria were used to define invasive strains;

1. All blood culture isolates

2. All stool isolates where blood culture was also positive

3. All stool isolates from patients presenting with fever > 39C and bloody diarrhoea or microscopy showing numerous (+++) leucocytes and red blood cells.

Non-invasive salmonellosis was defined as NTS from patients with stool culture, no fever, and watery diarrhoea and vomiting.

Consenting parents/caregivers to the children were asked to respond to a questionnaire seeking to identify where they came from, and their sources of food and drinking water. Homes were categorised as upper class residence, middle class, lower middle class or slum area, according to structure of property and socioeconomic status of family using guidelines from the Central Bureau of Statistics [26]; upper class residents owned/rented property that was brick walled and tiled roof and monthly income > $275; middle class residents lived in stone walled and tiled/iron sheet roofed and earned a monthly income of $90–$275; lower middle residents were either in timber walled houses with iron sheet roofs and earned a monthly income of $25–$90; slum area residents lived in mud-walled and tin/plastic roofed houses and earned less than $25 per month. The following data were also prospectively recorded: demographic, clinical, microbiologic features including bacterial genotypes and antibiotic susceptibility patterns of strains, and treatment offered. All specimens were taken before antibiotic treatment commenced and usually within 12 hours after admission. Research ethical approval was granted by the Ethical Review Boards of KEMRI and the Liverpool School of Tropical Medicine.

### Microbiologic methods

Blood cultures were processed using standard techniques. Briefly, blood cultures were incubated in 5% CO_2 _in air at 37°C for 18 h and if signs of bacterial growth were observed (air bubbles, turbidity or both) they were subcultured onto sheep blood agar and chocolate agar. The remaining blood cultures were reincubated for a further 7 days or until positive. Stools were processed by direct plating onto selective media (XLD and brilliant green agar) (Oxoid Ltd., Basingstoke, UK) and by overnight enrichment in selective Selenite F broth (Oxoid) followed by plating onto XLD and brilliant green agar (Oxoid), and incubated in air at 37°C for 18 h. NTS were identified using agglutinating antisera (Murex Biotech Ltd., Dartford, UK) and their identification was confirmed biochemically using API 20E strips (BioMerieux, Montalieu Vercieu, France).

### Antimicrobial susceptibility testing

Susceptibilities to antimicrobials – ampicillin, co-amoxiclav, tetracycline, cotrimoxazole, chloramphenicol, gentamicin, nalidixic acid, ciprofloxacin, cefuroxime and ceftriaxone – were determined by both controlled disk diffusion and measuring minimum inhibitory concentrations (MICs) using E-test strips (AB BIODISK, Solna, Sweden) according to the manufacturer's instructions. *Escherichia coli *ATCC 25922 and ATCC 35218 (with known MICs) were used as a control for potency of antibiotic disks and E-test strips. In addition, testing of susceptibility to ceftriaxone and to ceftazidime alone (E-test MIC strips; AB BioDisk, Solna, Sweden) and in combination with clavulanic acid (E-test extended-spectrum-TEM-and-SHV-β-lactamase strips) was performed to determine production of extended spectrum beta-lactamases by NTS strains. Disk diffusion susceptibility tests and MICs were interpreted according to the guidelines provided by the Clinical and Laboratory Standards Institute [27].

### Pulsed field gel electrophoresis of macrorestricted chromosomal DNA

Chromosomal DNA from NTS isolates was prepared in agarose plugs as described previously [10]. DNA in agarose plugs was digested using 25 units each of *Xba*I or *Spe*I (Roche Diagnostics GmbH, Mannheim, Germany). PFGE of agarose plug inserts was then performed on a CHEF-DR III system (Bio-Rad Laboratories, Hercules, Ca, USA) on a horizontal 1% agarose gel for 20 h at 120 V, with a pulse time of 1 to 40 s at 14°C. A lambda DNA digest consisting of a ladder (ca. 22 fragments) of increasing size from 50 kb to approximately 1000 kb was included as a DNA size standard. The gel was stained with ethidium bromide and photographed on an UV transilluminator (UVP Inc., San Gabriel, CA, USA). The Restriction Endonuclease digest patterns were compared and their similarities were scored using the Dice similarity coefficient formula, 2h/(a+b), where h is the number of matching bands, a+b is the total number of bands including matching and non-matching. Isolates that differed in their PFGE fragment patterns by one or two bands were regarded as closely related as minor mutational changes would result in such patterns.

### Statistical analysis

We compared proportions of characteristics in cases of NTS bacteraemia and those of NTS gastroenteritis by using the Chi-squared or Fisher's exact test. Continuous variables were compared by using the *t-*test or the Mann-Whitney *U *test.

## Authors' contributions

SK conceived the study, supervised molecular analysis and prepared manuscript. JK performed molecular typing. JM did preliminary microbiology. GR and NK recruited patients and were involved in care of patients. CAH was co-PI on grant and helped in analysis and manuscript preparation. All authors read and approved the final manuscript.

## Figures and Tables

**Table 1:** Characteristics of children with NTS stratified by blood and stool culture positivity

Characteristic [No. (%)]	Stool -ve blood +ve (N = 170)	Stool +ve Blood +ve (N = 28)	Stool +ve blood -ve (N = 134)	p-value
Sex				
Male	98 (57.6)	17 (60.7)	76 (57.6)	
Female	72 (42.3)	11 (39.3)	56 (42.4)	0.931
Mean age (months)	22	24	24	0.7681
Key Clinical features				
Fever	105 (61.8)	12 (42.9)	37 (27.6)	p < 0.001
Diarrhoea	69 (40.6)	26 (92.9)	89 (66.4)	p < 0.001
Vomiting	44 (28.9)	2 (7.1)	66 (49.3)	p < 0.001
Days hospitalised				
Range	1–60	1–30	1–23	-
Median	9	7	2	0.004
NTS serotypes				
*S*. Typhimurium	93 (54.7)	11 (39.3)	92 (68.7)	0.04
*S*. Enteritidis	57 (33.5)	8 (25)	29 (21.6)	0.71
Other serotypes	20 (11.8)	9 (32.1)	13 (9.7)	0.13

**Table 2:** NTS presentation according to classification of homes of index cases

	Residence and No. (%) of cases			
Presentation	Slum N = 128	Lower middle and middle class n = 123	Upper class n = 81	p-value
Diarrhoea alone	54(42.2)	57(46.4)	56(69.1)	p < 0.001
Diarrhoeal+ Invasive NTS	12(9.4)	19(15.4)	11(13.6)	p = 0.427
Invasive NTS alone	62(48.4)	47(38.2)	14(17.3)	p < 0.001

**Table 3:** MIC using the E-Test of 10 antimicrobial agents for 198 NTS isolates from blood cultures (BC) and 162 NTS isolates from stools (ST) at two Hospitals in Nairobi, Kenya (2002–2004).

Antimicrobial	Minimum Inhibitory Concentration (μg/ml)					
Agent	Range		MIC_90_		% Resistant	
	BC	ST	BC	ST	BC	ST
Ampicillin	0.25->256	0.25->256	64	64	54	49
Co-amoxiclav	0.75->256	0.38–32	16	16	8	13
Cefuroxime	2->256	2->256	32	32	30	28
Ceftriaxone	0.094–16	0.023–6	2	0.12	0	0
Gentamicin	0.06->256	0.125->256	8	6	16	9
Co-trimoxazole	0.064->32	0.032->32	32	32	46	48
Chloramphenicol	0.19->256	0.19->256	32	32	26	22
Tetracycline	0.064->256	0.75->256	128	32	51	39
Nalidixic acid	1.5->256	1.5->256	3	8	12	6
Ciprofloxacin	0.064–4	0.008–0.125	0.125	0.032	0	0

**Table 4:** Most common antibiotic treatment choices used for NTS infections.

	No (%) treated	Overall % Resistance
Cefuroxime	77 (32)	30
Ceftriaxone	64 (26.4)	0
Gentamicin	42 (17.4)	16
Co-amoxiclav	29 (12)	8
Ampicillin	21 (8.7)	48
Cotrimoxazole	8 (0.03)	46
